# Disentangling the aetiological pathways between body mass index and site-specific cancer risk using tissue-partitioned Mendelian randomisation

**DOI:** 10.1038/s41416-022-02060-6

**Published:** 2022-11-24

**Authors:** Genevieve M. Leyden, Michael P. Greenwood, Valérie Gaborieau, Younghun Han, Christopher I. Amos, Paul Brennan, David Murphy, George Davey Smith, Tom G. Richardson

**Affiliations:** 1 MRC Integrative Epidemiology Unit, Bristol Population Health Science Institute, University of Bristol, Bristol, BS8 2BN UK; 2 Bristol Medical School: Translational Health Sciences, Dorothy Hodgkin Building, University of Bristol, Bristol, BS1 3NY UK; 3 Genomic Epidemiology Branch, International Agency for Research on Cancer (IARC/WHO), Lyon, France; 4 Institute for Clinical and Translational Research, Baylor College of Medicine, Houston, TX USA; 5 Section of Epidemiology and Population Sciences, Department of Medicine, Baylor College of Medicine, Houston, TX USA; 6 Dan L Duncan Comprehensive Cancer Center, Baylor College of Medicine, Houston, TX USA

**Keywords:** Gene expression, Cancer, Cancer epidemiology, Cancer genetics, Risk factors

## Abstract

**Background:**

Body mass index (BMI) is known to influence the risk of various site-specific cancers, however, dissecting which subcomponents of this heterogenous risk factor are predominantly responsible for driving disease effects has proven difficult to establish. We have leveraged tissue-specific gene expression to separate the effects of distinct phenotypes underlying BMI on the risk of seven site-specific cancers.

**Methods:**

SNP-exposure estimates were weighted in a multivariable Mendelian randomisation analysis by their evidence for colocalization with subcutaneous adipose- and brain-tissue-derived gene expression using a recently developed methodology.

**Results:**

Our results provide evidence that brain-tissue-derived BMI variants are predominantly responsible for driving the genetically predicted effect of BMI on lung cancer (OR: 1.17; 95% CI: 1.01–1.36; *P* = 0.03). Similar findings were identified when analysing cigarettes per day as an outcome (Beta = 0.44; 95% CI: 0.26–0.61; *P* = 1.62 × 10^−6^), highlighting a possible shared aetiology or mediator effect between brain-tissue BMI, smoking and lung cancer. Our results additionally suggest that adipose-tissue-derived BMI variants may predominantly drive the effect of BMI and increased risk for endometrial cancer (OR: 1.71; 95% CI: 1.07–2.74; *P* = 0.02), highlighting a putatively important role in the aetiology of endometrial cancer.

**Conclusions:**

The study provides valuable insight into the divergent underlying pathways between BMI and the risk of site-specific cancers.

## Introduction

Body mass index (BMI) is an important risk factor for multiple types of cancer. Mendelian randomisation (MR) [1] studies have been integral in elucidating evidence of causal relationships between variation in BMI and site-specific cancer risk [2], although further granular insight is required to clarify the specific mechanistic and biological pathways which may explain these effects. While BMI is a commonly used proxy for excess adiposity in population studies, it retains a high degree of heterogeneity and therefore captures multiple phenotypes [3].

Recently, we have developed a novel multivariable MR approach to separate the effects of phenotypic subcomponents of BMI on complex traits and disease risk. This is implemented through fractionation of the genetic variants associated with BMI according to whether the BMI signal colocalises with gene expression in the brain or subcutaneous adipose tissue [4]. In this framework, ‘adipose-’ and ‘brain-tissue instrumented BMI’ were analysed as separate exposures in a one-sample multivariable MR analysis using genetic risk scores (GRS) based on subsets of adipose and brain expression colocalizing BMI variants. We found that these distinct tissue-dependent exposures related differentially to measures of fat distribution and visceral adiposity, with brain-tissue colocalizing variants driving the effect of BMI on cardiometabolic disease outcomes and subcutaneous adipose-tissue colocalizing variants predominantly being responsible for the effect of BMI on measures of heart structure [4].

In this study, we sought to adapt this tissue-partitioned MR approach such that it can be applied in a two-sample MR setting, allowing us to leverage findings from large consortia. Next, we applied this approach to investigate the putatively independent effects of adipose- and brain-tissue instrumented BMI on the risk of seven site-specific cancer outcomes. Lastly, we conducted further analyses using additional datasets to evaluate the robustness of potential independent effects highlighted in our primary analysis.

## Methods

### Tissue-partitioned genetic instruments for adult BMI

Full details on the genetic instruments identified for MR analyses in this study have been reported previously [4] and are explained in further detail in Supplementary Note 1. In brief, we incorporated gene expression data to identify genetic variants whose robust effects on BMI are putatively mediated via the expression of a nearby gene in either subcutaneous adipose and neural tissues. This was assessed by conducting extensive genetic colocalization analyses using the method ‘*coloc’* [5] where a posterior probability for colocalization (PPA4) ≥0.8 was applied to formally define instrument sets, as recommended by the authors of the method.

Genetic colocalization analyses were performed systematically at 915 independent loci robustly associated with adult BMI (i.e., *P* < 5 × 10^−08^ & *r*^2^ < 0.01) from a meta-analysis GWAS from the Genetic Investigation of Anthropometric Traits (GIANT) consortium and the UK Biobank (UKB) (*n* = 681,275) [6]. To ensure that the highest SNP coverage available was implemented for colocalization analyses, we combined these data with the summary statistics from a BMI GWAS involving participants of European ancestry from the UK Biobank only (*n* = 463,005) to obtain summary statistics for SNPs not included in the meta-analysis with GIANT. The combined BMI datasets provided summary statistics on a total of 12,322,387 SNPs. To minimise the incorporation of findings which may potentially be influenced by strong regional linkage disequilibrium (LD) structure we omitted variants which reside within the human leukocyte antigen (HLA) region (chr6:25 Mb–35 Mb).

In total, 86 genetic variants provided evidence of colocalization between BMI and proximal subcutaneous adipose-tissue-derived gene expression using meta-analysed expression quantitative loci (eQTL) data derived from subcutaneous adipose (*n* = 1257). Similarly, 140 genetic variants with evidence of colocalization were found between BMI and proximal brain-tissue-derived gene expression data using meta-analysed brain-tissue samples (*n* = 1194). These two instrument sets were used as proxies for what we refer to as 'adipose-' and 'brain-tissue instrumented BMI', respectively. These tissues were selected due to their important biological relevance for adiposity and resulting instruments were subject to various robustness evaluations such as ensuring that both resulting instrument sets have very similar average effect estimates on BMI (adipose = 0.0148 and brain = 0.0149 standard deviation change in BMI per effect allele) (Supplementary Note 1). Full details of the genetic variants incorporated into adipose- and brain-tissue instrumented BMI exposures are provided in Supplementary Table S1.

Robust simulation studies in the literature have suggested that sample sizes of eQTL studies over *n* = 1000 should maintain a high true positive and low false-positive rate for the majority of common variants identified by GWAS [7]. We additionally assessed how the number of instruments may influence exposure strength in the multivariable model. Randomly sampling pools of adipose- and brain-tissue instruments suggested that even when 30 BMI instruments for both tissues are available our multivariable approach is capable of separating these two exposures (F_adipose_ = 30.6 & F_brain_ = 29.9).

### Tissue-partitioned childhood body-size instruments

In this study, we additionally applied our instrument derivation pipeline as described above to GWAS results from a measure of childhood body size using recall data from the UK Biobank study at age 10 [8]. UKB participants completed recall questionnaires asking if they were ‘thinner’, ‘plumper’ or ‘about average’ when they were aged 10 years old compared to the average. These GWAS results have been previously validated using measured childhood BMI in three independent cohorts which found that they predict BMI at this early stage in the lifecourse more strongly compared to adult BMI genetic variants [9–11]. In addition, the childhood BMI phenotype has been shown to directly influence outcomes measured during childhood such as vitamin D levels [12], but have an indirect effect after accounting for genetically predicted adulthood adiposity on the same outcome when measured in adulthood. There were 56 and 53 childhood body-size-associated variants which colocalized with adipose- and brain-tissue eQTL, respectively. A list of these genetic variants can be found in Supplementary Table S2.

### Genome-wide association study data on site-specific cancers

GWAS estimates were obtained on the following seven site-specific cancer outcomes: colon, breast, endometrial, lung, ovarian, kidney and prostate cancer [13**–**17]. All site-specific cancers investigated in the present analysis have previously been shown to be causally influenced by adiposity in MR analyses [18**–**20]. If a particular SNP was not present in the outcome summary data extracted from the MRC-IEU OpenGWAS database using MR-base [21, 22], a proxy SNP in LD with the requested SNP was provided by default. LD proxies were determined using the 1000 genomes of European ancestry sample data. To maximise statistical power for cancer outcomes where reported parental history of disease in the UK Biobank study provided a larger number of cases compared to accessible datasets (i.e., colon and lung cancer), we obtained estimates based on a GWAS by proxy approach previously shown to be highly genetically correlated with findings from GWAS of diagnosed cases [23, 24]. A summary of the outcome datasets used in this study is provided in Supplementary Table S3.

### Univariable Mendelian randomisation to estimate the total effect of BMI

We firstly estimated the total effect of genetically predicted adult BMI using the full set of 915 instruments (i.e., without considering their tissue-dependent effects on gene expression) on the seven site-specific cancer outcomes which have previously been shown to be influenced by adiposity [18**–**20]. Analyses were conducted using two-sample Mendelian randomisation (MR) with the inverse-variance weighted (IVW) method [25] and repeated using the MR Egger, weighted median and MR penalised weighed median methods, which are typically more robust to horizontal pleiotropy [26]. All analyses were conducted using the ‘*TwoSampleMR*’ R package. Estimates for instruments when analysing cancer endpoints based on GWAS of sex-stratified populations (i.e., breast, endometrial and ovarian cancers in female-only populations, prostate cancer in a male-only population) were obtained from previously conducted sex-stratified GWAS analyses of BMI [27]. MR analyses were conducted using exposure and outcome data from non-overlapping samples where possible to avoid overfitting bias [28].

### Tissue-partitioned Mendelian randomisation

Next, we used the sets of adipose and brain expression variants which colocalized with BMI based on PPA4 ≥ 0.8 as instrumental variables within the MR framework. When using the adipose colocalized variants as genetic proxies for BMI, we refer to this exposure as ‘adipose-tissue instrumented BMI’ hereafter, whereas when using the subset of brain colocalized variants as instruments, we refer to this exposure as ‘brain-tissue instrumented BMI’. We conducted univariable MR as above to estimate the total effect of adipose and brain-expressed BMI separately on all seven site-specific cancers.

We next employed a multivariable MR (MVMR) approach to estimate the direct effects of these tissue-partitioned exposures on each outcome by simultaneously estimating their effects in the same model. We previously demonstrated the use of MVMR to separate the effects of adipose- and brain-tissue instrumented BMI in a one-sample MR setting for various cardiovascular disease traits [4]. Due to the current limited availability of individual-level data with large numbers of cancer cases, we adapted the methodology to leverage GWAS summary statistics for which there are publicly available data from highly powered meta-analyses studies conducted by consortia. Simulations were conducted using the ‘*simulateGP*’ R package to evaluate the relative power of this approach across a range of effect sizes (0.1, 0.125 and 0.15), outcome sample sizes (10,000, 25,000, 50,000, 75,000 and 100,000) and proportion of variance explained by tissue-partitioned instruments (0.5%, 1%, 1.5%, 2%, 2.5% and 3%) derived from a simulated GWAS of *n* = 700,000 with a pool of 915 independent genetic instruments (based the BMI GWAS by Yengo used in our applied analysis).

**Fig. 1:**
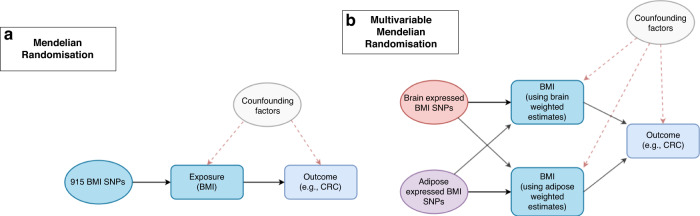
Schematic diagram of Mendelian randomisation (MR) analyses. The total effect of BMI (**a**) was estimated using univariable MR analysis. The independent effect of adipose- and brain-tissue instrumented BMI was estimated using a multivariable MR approach (**b**) by weighting the beta effect estimates of the SNP-exposure associations by their PPA4 values assessed by colocalization for each tissue respectively.

The independent effects of adipose- and brain-tissue instrumented BMI were estimated using MVMR by weighting the beta effect estimates of the SNP-exposure associations by their PPA4 values assessed by colocalization for each tissue, respectively. This weighting scheme was devised to incorporate the evidence that genetic instruments putatively influence BMI due to their expression in either adipose or brain tissues (i.e., SNPs with a very small PPA4 value were down-weighted using our approach as they are unlikely to influence BMI via gene expression in adipose or brain tissue). A schematic diagram of this approach is illustrated in Fig. 1.

We next applied our novel two-sample MVMR approach to investigate whether the effect of BMI on the seven cancer types is predominantly attributed to genetic variants exerting their effects via subcutaneous adipose- or brain-tissue-related pathways. Importantly, we emphasize that the effect estimates derived using tissue-partitioned instruments should not be interpreted as causal effects in the same manner as more conventional risk factors when analysed using MR. Instead, we have developed this approach to investigate the separate contributions of genetic instruments that relate to different forms of a given trait, applied in this study using BMI and adipose/brain-tissue-derived gene expression as an exemplar. To this end, our framework provides a novel approach to dissect disease pathways between risk factors and endpoints by leveraging genetic instruments under the principles of MR.

In addition, we assessed the sensitivity of this weighted two-sample MVMR approach by analysing the same cardiovascular disease endpoints and measures of cardiac structure investigated in our previous study [4] (Supplementary Tables S4 and S5). Doing so suggested that the more widely applicable two-sample approach was capable of recapitulating findings from its application in a one-sample MVMR setting (Supplementary Note 2). Instrument strength in MVMR analyses was evaluated using conditional F-statistics as derived with the *‘MVMR’* R package with F > 10 used as an indication that weak instrument bias was not influencing our findings [29] (Supplementary Table S6). Conditional F-statistics were particularly important to evaluate within our MVMR framework to demonstrate that the two molecular forms of BMI being analysed could be instrumented as two separate exposures in our model. In order to maximise the number of reliable instruments incorporated in exposure variables, we demonstrated the effect of varying the PPA threshold for eQTL instrument identification on conditional F-statistics in an analysis on coronary artery disease (Supplementary Fig. S1). Lowering the PPA threshold in our inclusion criteria resulted in weaker instruments as indicated by their conditional F-statistics. We therefore advocate that the recommended PPA4 threshold proposed by the method developers (i.e PPA4 > 0.8) be used when identifying tissue-partitioned instruments.

## Results

### Univariable MR analyses of BMI effects on cancer outcomes

MR analyses were first carried out to estimate the total effect of genetically predicted BMI using all 915 independent variants and each of the 7 cancer endpoints. The MR estimates reproduced findings from previously published MR studies of the relationship between adiposity and site-specific cancers (i.e., without considering tissue-dependent effects on gene expression) [18**–**20] (Supplementary Table S7 and Supplementary Fig. 2a). Univariable MR analyses of BMI instrumented with the adipose- and brain-tissue colocalized variants found strong evidence of an effect on the risk of outcomes such as endometrial (adipose: OR = 1.84; 95% CI = 1.35–2.51; *P* = 9.88 × 10^−5^; brain: OR = 1.61; 95% CI = 1.32–1.97; *P* = 3.7 × 10^−6^) and lung (adipose: OR = 1.07; 95% CI = 0.95–1.19; *P* = 0.27; brain: OR = 1.17; 95% CI:1.07–1.27; *P* = 0.0003) cancer. Evidence of a genetically predicted effect using tissue-partitioned BMI was also found on a lower risk of prostate cancer (adipose: OR = 0.79; 95% CI = 0.6–0.98; *P* = 0.03; brain: OR = 0.77; 95% CI = 0.65–0.91; *P* = 0.002).

**Fig. 2:**
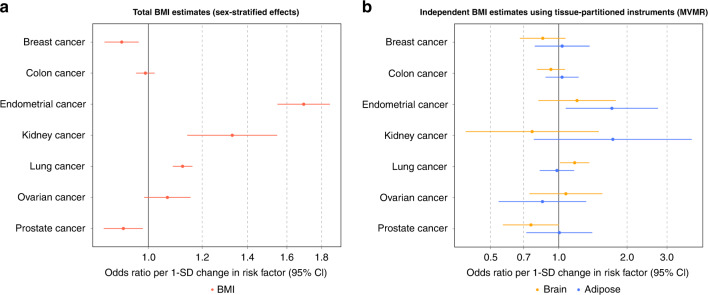
Forest plot summarising the results of Mendelian randomisation analyses. Summary of Mendelian randomisation results for BMI on 7 site-specific cancers based on (**a**) univariable analyses using the total set of BMI variants and (**b**) analyses instrumented in a multivariable setting with tissue-partitioned variants. Forest plots illustrating the odds ratios per change in risk factor and 95% confidence intervals (CIs) for each outcome analysed by MR are shown. The effect estimates of BMI instrumented with all 915 BMI SNPs is illustrated in (**a**) (red), and the independent effect estimates of BMI instrumented by adipose- (blue) and brain (orange)-tissue-derived instruments in the multivariable MR model are illustrated in (**b**).

### Multivariable MR analyses of tissue-partitioned BMI effects on cancer outcomes

Applying our weighted two-sample MVMR approach to separate the ‘independent’ effects of adipose- and brain-tissue instrumented BMI highlighted instances where these tissue-partitioned sets of variants may contribute differentially to site-specific cancer risk (Fig. 2). For example, the independent effect of brain-tissue instrumented BMI on risk of endometrial cancer attenuated when analysed simultaneously with adipose-tissue instrumented BMI (OR = 1.20; 95% CI = 0.81–1.78; *P* = 0.36), whereas the effect of adipose-tissue instrumented BMI remained strong (OR = 1.71; 95% CI = 1.07–2.74; *P* = 0.02). Conversely, evidence of an independent effect of adipose-tissue instrumented BMI on lung cancer risk attenuated in the MVMR model (OR = 0.98; 95% CI = 0.82–1.17; *P* = 0.83) and the effect of brain-tissue instrumented BMI remained strongly positive (OR = 1.17; 95% CI = 1.01–1.36; *P* = 0.03). A comparison of all univariable and multivariable estimates for each site-specific cancer outcome is provided in Supplementary Table S8.

Weak evidence of an independent effect was detected for both adipose- (OR = 1.03; 95% CI = 0.78–1.37; *P* = 0.80) and brain-tissue (OR = 0.85; 95% CI = 0.67–1.07; *P* = 0.17) instrumented BMI and breast cancer. Given the emerging role of childhood obesity in breast cancer risk [9], we re-applied our entire instrument derivation pipeline using results from a large-scale GWAS of childhood body size based at age 10 in the lifecourse. In total, 56 variants provided strong evidence of colocalization with proximal gene expression derived from subcutaneous adipose tissue, and 53 variants using gene expression data from brain-derived tissue (Supplementary Table S2). The mean absolute effect for each subset of these tissue-partitioned instruments on childhood body size were similar (adipose = 0.013, brain = 0.013). Although both adipose- and brain-tissue instrumented childhood body size effects provided evidence of an effect on breast cancer risk in a univariable setting (adipose: OR = 0.59; 95% CI = 0.41–0.87; *P* = 0.007; brain: OR = 0.58; 95% CI = 0.42–0.81; *P* = 0.001), only weak evidence of an independent effect was found in the multivariable MR analysis for adipose-tissue instrumented childhood body size (OR = 0.98; 95% CI = 0.55–1.73; *P* = 0.93). In contrast, the central estimate for genetically predicted childhood body size when instrumented using brain-tissue colocalized variants remained robust in a multivariable setting (OR = 0.57; 95% CI: 0.33–0.98; *P* = 0.04) (Supplementary Table S9).

### Replication and negative control analyses

As a further analysis, we incorporated additional datasets to investigate the replicability of the findings for endometrial and lung cancer, and we advocate similar validation analyses for any future applications of our MVMR approach. The independent effect of adipose-tissue instrumented BMI on endometrial cancer risk is supported by an analysis using data obtained from the UK Biobank (UKB) and the Kaiser Permanente Genetic Epidemiology Research on Adult Health and Aging (GERA) cohorts [30] (OR = 3.03; 95% CI: 1.42–6.47; *P* = 0.004), despite the small case numbers in this dataset. We were unable to replicate the independent effects of brain-tissue instrumented BMI on lung cancer using an additional case–control GWAS study [31] (OR = 1.10; 95% CI: 0.78–1.55; *P* = 0.57). However, we were able to provide evidence that brain-tissue instrumented BMI is predominantly responsible for driving the relationship between BMI and ‘cigarettes smoked per day’ when analysed as an outcome (adipose: Beta = 0.03; 95% CI = –0.18–0.24; *P* = 0.76; brain: Beta = 0.44; 95% CI = 0.26–0.61; *P* = 1.62 × 10^−6^) (Supplementary Table S8), which is noteworthy given the strong causal effect that smoking has on lung cancer risk. Detailed results of all replication analyses are provided in Supplementary Table S10.

As a final sensitivity analysis, we repeated our entire instrument derivation pipeline using gene expression data from tissues which are unlikely to be biologically relevant for the BMI-associated genetic variants (e.g., minor salivary gland and ovary tissues [32, 33] (Supplementary Note 3)). In contrast to findings from our primary analysis, there was very weak evidence to suggest that partitioning variants from these putatively non-causal tissues for BMI leads to robust evidence of an effect on the site-specific cancer endpoints analysed previously (Supplementary Table S11). In addition, we sought to evaluate the effects where adipose- and brain-tissue instruments provided evidence of an independent effect on cancer outcomes using data from the eQTLGen consortium [34] (*n* = 31,684) to assess the sensitivity of the findings to gene expression data derived from whole blood that may not capture tissue-specific effects (Supplementary Note 3). The independent effect of brain-instrumented BMI on lung cancer risk remained robust when analysed simultaneously with whole blood instrumented BMI (OR = 1.14; 95% CI = 1.00:1.30; *P* = 0.04), while the whole blood BMI effect attenuated (OR = 1.09; 95% CI = 0.96:1.23; *P* = 0.18). Similarly, the independent effect of adipose-instrumented BMI on endometrial cancer replicated when analysed simultaneously with whole blood instrumented BMI (OR = 2.33; 95% CI = 1.06:5.12; *P* = 0.03), while weak evidence of an independent effect was obtained for whole blood instrumented BMI (OR = 1.07; 95% CI = 0.58:1.96; *P* = 0.83) (Supplementary Table S12).

Lastly, we attempted to partition genetic instruments using the adipose- and brain-tissue-derived datasets used in our primary analysis for a phenotype where these tissues are unlikely to be functionally important. We selected a GWAS of psoriasis for this purpose previously conducted in the UKB (*n* = 462,933) which further reinforced that tissue types need to be carefully selected for our approach to produce meaningful results, given that we identified only 2 variants with evidence of colocalization with adipose-tissue-derived gene expression and only 1 variant with brain-derived gene expression (Supplementary Note 3).

## Discussion

The role of obesity in cancer aetiology is highly complex. In this study, we have applied the principles of MR to estimate the effects of separate tissue-partitioned subcomponents of BMI on the risk of seven site-specific cancer outcomes which have previously been shown to be influenced by adiposity [18**–**20]. We firstly demonstrated that the results generated using our two-sample MVMR approach to detect distinct adipose- and brain-tissue BMI-mediated effects provide concordant results with a recently conducted one-sample multivariable MR analysis on cardiovascular disease and cardiac structure phenotypes [4]. Application of this novel extension of multivariable MR to cancer outcomes provides mechanistic insight into the distinct pathways underlying variation in BMI and risk of developing certain cancer types, particularly endometrial, lung and breast cancer.

Endometrial cancer is more strongly associated with obesity than any other cancer [35, 36]. Adipose-tissue accumulation is an important driver of endometrial cancer progression via three main mechanisms: excess oestrogen exposure [37, 38], insulin resistance [39], and the induction of pro-inflammatory phenotypes as a result of hypoxia following adipose-tissue expansion [40, 41]. The variation in gene expression captured by the subcutaneous adipose-tissue instrumented BMI exposure in this study may have several molecular consequences which can be postulated to differentially influence disease aetiology. For instance, adipose tissue is a major source of multipotent mesenchymal stem cells (MSCs) which have significant proliferative capacity [42, 43]. The characteristic migration of MSCs towards sites of injury [44, 45] includes the sites of several tumour types and has been shown to contribute to cell growth in tumour microenvironments [46**–**48]. Regional differences in proliferation and differentiation may favourably impact metabolic phenotypes, explaining the attenuated effect observed between adipose-BMI on common obesity comorbidities such as T2D [4, 49, 50]. A plausible explanation for this has been attributed to variation in fat distribution, whereby adiposity-increasing alleles are associated with a greater capacity to store fat subcutaneously as opposed to viscerally are protective [50].

On the other hand, our results suggest that adipose-tissue BMI may capture a particular phenotype which is more susceptible to inducing endometrial tumorigenicity. Genetic loci incorporated into the adipose-tissue BMI exposure included several regulators of adipogenesis which may be of prognostic importance for endometrial cancer. For example, *FST* encodes the adipokine follistatin which has been shown to regulate adipocyte differentiation [51**–**53] and is also a marker of polycystic ovary syndrome (PCOS) [54]. The developmental transcription factor *TBX15* influences adipogenesis [55**–**57] and has recently been identified as a key regulator of co-expression networks regulating central adiposity [58]. Furthermore, the expression of *CADM1* has a role in extracellular matrix adhesion [59] and has been shown to promote endometrial cancer progression [60**–**62]. These findings help to establish an important link between the regulation of body composition and endometrial cancer risk and warrant further functional investigation.

The epidemiological evidence for the relationship between obesity and lung cancer is complicated, with some studies reporting seemingly paradoxical findings [63**–**66]. This can most likely be attributed to the strong potential for confounding caused by smoking status and the effect of smoking on body weight. Our results are consistent with a positive causal effect of genetically predicted higher BMI on lung cancer risk, which has been reported in earlier MR studies [18, 19]. The results of the multivariable MR analysis suggest that BMI when instrumented using the brain-tissue BMI exposure independently increases risk of lung cancer when the effect of adipose-tissue BMI is accounted for. This is further supported by the independent effect observed for brain-tissue BMI on cigarette smoking shown in our sensitivity analysis. A potential limitation of the analysis to detect supporting evidence of the brain-tissue instrumented BMI effect in our replication dataset may have been introduced by covariate adjustment amongst the contributing consortia for variables including alcohol dependence. Investigating the effect of our tissue-partitioned instruments on alcohol intake frequency suggests that the brain-tissue-derived variants relate more strongly to this behavioural trait (Supplementary Table S8). As such, this finding therefore requires further investigation by future studies.

Previously, we postulated that the brain-tissue BMI exposure may relate to a molecular phenotype which predominantly influences BMI through a genetic predisposition for increased adiposity, likely arising from variation in appetite and energy intake [4]. In addition, our findings contribute to our understanding of the positive relationship between BMI and smoking. BMI has been shown to bi-directionally associate with smoking [67]; whereby having a higher BMI is positively associated with smoking [67**–**69], and smoking heaviness inversely effects BMI [70**–**72]. The overall positive relationship between BMI and smoking behaviour has been well replicated, likely influenced both by behavioural [73**–**76] and physiological factors [77], while the inverse relationship between smoking heaviness and BMI may be mediated by the effect of nicotine on energy balance [2]. The brain-tissue BMI exposure reflects the BMI phenotype leading to smoking, suggesting that smoking may be a mediator between BMI and lung cancer, or may be partly influenced by a shared aetiology for BMI and smoking [78, 79]. For example, adiposity genes incorporated into the brain-tissue BMI exposure such as *BDNF* and *OPRL1*, have each been shown to contribute to energy intake [80**–**82], binge eating [83**–**85], and smoking initiation [86**–**89]. Furthermore, association studies have identified a positive relationship between sensitivity to sweet-tasting stimuli and impulsive behaviour [90]. Among the loci incorporated into the brain-tissue BMI exposure are several genes which are highly represented within the sweet taste signalling pathway (e.g., *KCNK3*, *PLCD4*, *PRKCD*) [91]. Taken together, these findings highlight compelling parallels between the impact of variation in neuroregulatory pathways on energy intake, smoking behaviour, and lifetime risk of lung cancer which will be important to delineate further.

Our univariable MR results align with studies which have established strong evidence indicating that a larger body size in childhood is protective against breast cancer risk [9, 92, 93]. The results of the multivariable MR suggest that the independent effect of childhood body size when instrumented using the brain-tissue BMI exposure may contribute to the protective effect on breast cancer. Nutritional status and higher adiposity in childhood are important drivers of earlier pubertal onset [94, 95], which is a demonstrated risk for breast cancer [96, 97]. Further exploration of the molecular characteristics of higher childhood BMI phenotypes on key developmental stages, such as age of menarche, may provide important insight on potential preventative measures. Previous MR studies [18, 93], including the results presented here, have also reported an inverse relationship between lifetime BMI and breast cancer risk. However, observational studies have suggested that higher adiposity is an important driver of breast cancer susceptibility in post-menopausal women [36, 98, 99]. As such, additional analyses stratified by pre- and post-menopause are needed to further investigate the independent effects of BMI via distinct tissue types on breast cancer risk.

This study has noteworthy limitations. In all MR analyses, a null effect was observed for the relationship between BMI and colon cancer (based on a GWAS by proxy study). While GWAS ascertained from a family history of the disease has demonstrated utility [23, 100], these resources are liable to have attenuated effect sizes and reduced statistical power relative to conventional GWAS datasets. Repeated analyses will be needed to determine these effects should the summary statistics from large-scale cohort studies on colon cancer become publicly available. Similarly, we do not report evidence for independent effects of adipose- or brain-tissue BMI on kidney cancer. Simulations suggest that our approach is adequately powered as long as tissue-partitioned instruments explained at least 1% of the variance in the exposure trait, as well as analysing outcome GWAS datasets based on at least 75,000 participants (Supplementary Fig. S2). As such, our approach should be repeated to evaluate the independent effects of tissue-partitioned instruments on kidney cancer once sufficient sample sizes become accessible. Furthermore, another important aspect to address in future studies will be the different aetiological subtypes of several of the cancer types assessed in the present study. For example, BMI is heterogeneously associated with the development of the histological subtypes of renal cell carcinoma (RCC) [101, 102], which may potentially influence attenuation of the observed associations between the tissue-stratified BMI exposures and kidney cancer. Lastly, the present study is focused on the effects of BMI mediated predominantly by neural and subcutaneous adipose gene expression, due to both sample size availability and biological relevance to BMI. Future analyses incorporating gene expression data from additional tissue types will likely yield further insight on important aetiological effects for site-specific cancers once sufficiently powered datasets are available.

In summary, we have demonstrated a novel application of multivariable MR which allowed us to investigate the genetically predicted effects of distinct molecular subcomponents of BMI on the risk of site-specific cancers. By extending this approach into a two-sample setting, we envisage that this will have wide applicability on a spectrum of disease outcomes where individual-level data obtained in highly powered cohort studies is not currently publicly available. Furthermore, our findings provide important insight into the divergent underlying pathways between body mass index and risk of site-specific cancers.

## Supplementary information

Reproducibility checklist

Supplementary Material

Supplementary Tables

## Data Availability

All summary-level data analysed in this study is publicly available, with the exception of the kidney and lung cancer GWAS data used for replication analysis. These can be accessed via a request to Dr Paul Brennan and Dr Chris Amos, respectively.
