# Supplementary material for: Disentangling the aetiological pathways between body mass index and site-specific cancer risk using tissue-partitioned Mendelian randomisation
Source: Br J Cancer. 2022 Nov 24;128(4):618–25. doi: 10.1038/s41416-022-02060-6 (PMC9938133; doi:10.1038/s41416-022-02060-6)
Supplement: Supplementary file 2 — Supplementary Material [file 41416_2022_2060_MOESM2_ESM.docx]

## Supplementary Note 1

Leyden et al

Summary statistics for BMI were obtained from a meta-analysis comprising data from the Genetic Investigation of ANthropometric Traits (GIANT) consortium and the UK Biobank (UKB) study (1). To ensure the highest available SNP coverage was integrated into the colocalization analysis, the summary data for SNPs not included in the meta-analysis were additionally combined with a separate GWAS of the UKB (n=463,005). 915 independent SNPs associated with BMI (P<5x10^-8^) were identified by applying linkage disequilibrium (LD) clumping using the PLINK software (2) based on a reference panel of data on 10,000 unrelated individuals in the UKB of European ancestry (r^2^<0.01) (3).

Meta-analyzed brain eQTL data was obtained from a study conducted by Qi et al (4) which included data from the GTEx consortium v7 (5), the CommonMind Consortium (CMC) (6) and the Religious Orders Study and Memory and Aging Project (ROSMAP) (7), total n=1194. A meta-analysis of subcutaneous adipose tissue derived eQTL data was performed using the MeCS method in the SMR package with summary from the MuTHER study (n=766) (8) and individuals of European ancestry in the GTEx consortium v8 (9) (n=491) (total n=1257). Adipose eQTL variant data was mapped to the hg19 genome-build using the GRCH37 reference assembly. The MuTHER and GTEx eQTL probes were harmonized using Ensembl Gene mappings prior to meta-analysis. Non-autosome and non-protein coding genes as defined by Ensembl were omitted from downstream analyses. Summary-level adipose eQTL data was converted to SMR (Summary Mendelian Randomization) input format using the ‘*gwas-summary*’ function in the SMR software package (v1.03) (10).

Genetic colocalization analyses were performed using the ‘*coloc*’ method with default parameters to evaluate the posterior probability (PPA) for colocalization between the 915 independent BMI GWAS SNPs based on LD clumping and the expression of proximal genes within a 200kb window. The ‘*coloc’* method estimates the posterior probability of five competing hypotheses: no association with either trait (PPA0); association with one trait (PPA1, PPA2); association with both traits but with distinct causal variants (PPA3); association with both traits with a common causal variant (PPA4) (11). Colocalization analyses were conducted twice at each locus; using eQTL data derived from the adipose tissue meta-analysis and then repeated separately using the eQTL data from brain tissue. A PPA4 >0.8 was considered strong evidence of colocalization, as recommended by the authors of the method.

These two sets of tissue-partitioned variants had a similar average magnitude of effect on BMI (adipose=0.0148 and brain=0.0149 standard deviation change in BMI per effect allele), although they related differently to alternative measures of anthropometry and adiposity. For instance, we previously found that the brain-tissue instruments were more strongly correlated with waist-to-hip ratio compared to the adipose-tissue instruments (r=0.733 & r=0.445 respectively, P_comparison_=0.001). Likewise, the brain-tissue set was more highly correlated than the adipose set with a measure of visceral adipose tissue from the UK Biobank study (r= 0.554 & r= 0.254 respectively, P_comparison_= 0.009), further suggesting that the way in which these tissue-partitioned sets of variants exert their effects on BMI likely varies in terms of biological pathways. An investigation of pathway enrichment amongst the genetic loci which yielded evidence for colocalization with either tissue also showed evidence for differential pathway enrichment (12). In general, pathways related to metabolic processes were enriched amongst the loci incorporated into the adipose-BMI exposure while more varied cellular processes were enriched amongst the loci incorporated into the brain-BMI exposure.

## Supplementary Note 2

Replication of CVD results

To demonstrate the sensitivity of the adapted two-sample MVMR approach, we first attempted to replicate previously published findings by analysing the adipose- and brain-instrumented BMI exposures against 6 cardiovascular disease outcomes and 4 measures of cardiac structure (12). The genetically predicted effect of BMI without taking their tissue-dependent effects on gene expression into account (i.e. using all 915 BMI variants as instrumental variables) provided strong evidence of an increasing effect on all outcomes except left ventricular ejection fraction (LVEF) (Beta=-0.06; 95% CI: -0.10- -0.02; P=0.005) in a two-sample MR analysis (**Table S10**). Overall, the results of the one-sample and two-sample MVMR analyses investigating adipose- and brain-instrumented BMI were concordant in terms of the predominant tissue-dependent exposure (**Table S11**). For example, the genetically predicted effect of BMI on risk of cardiovascular disease outcomes such as type 2 diabetes (T2D) (OR=2.38; 95% CI=1.41-3.99; P=0.001), coronary heart disease (CHD) (OR=1.43; 95% CI=1.08-1.91; P=0.01) and heart failure (HF) (OR=1.60; 95% CI=1.26-2.05; P=0.0001) remained strong for brain-tissue instrument BMI when the effect of adipose-tissue instrumented BMI was accounted for in the same model using the two-sample MVMR approach. Additionally, the adipose-tissue instrumented BMI effect on cardiovascular disease outcomes attenuated when accounting for the effect of brain-tissue instrumented BMI (e.g.: T2D (OR=0.81; 95% CI=0.44-1.50; P=0.50), CHD (OR=1.18; 95% CI=0.84-1.66; P=0.34), HF (OR=1.23; 95% CI=0.92-1.64; P=0.16)).

Conversely, the opposite trend was observed for measures of cardiac structure as identified previously in a one-sample setting. Structural changes in the heart, for example increases in left ventricular end-diastolic volume (LVEDV) and left ventricular end-systolic volume (LVESV), are a defining feature of dilated cardiomyopathy and an important predictor of HF. The positive relationship between BMI and left-ventricular cardiac measures such as LVEDV (Beta=0.23; 95% CI=0.02-0.43; P=0.03) and SV (Beta=0.23; 95% CI=0.02-0.45; P=0.04) was maintained by the adipose-tissue instrumented BMI exposure, while the brain-tissue instrumented BMI effect attenuated (LVEDV: Beta=0.06; 95% CI:-0.12-0.23; P=0.51; SV: Beta=0.07; 95% CI:-0.11-0.26; P=0.43), as reported previously (12).

## Supplementary Note 3

Negative control analysis using ‘null’ tissues and exposure

To demonstrate the importance of carefully selecting biologically relevant tissue types when partitoning genetic instruments for an exposure using our approach, we repeated our entire instrument derivation pipeline using tissues which are unlikely to be of biological relevance for BMI. We obtained eQTL from minor salivory gland and ovary tissues from the GTEx Consortium (v8) (9) for this purpose as the derived sets of instruments provided very similar average magnitudes of effect on BMI (minor salivery gland=0.0147 and ovary=0.0148 per 1-SD change in BMI). Similarly, the resulting instruments sets (n=33.6 variants for minor salivory gland and n=45.1 variants for ovary) yielded conditional F-statistics which suggested that they were unlikely to be prone to weak instrument bias. This was not the case when evaluating other tissue combinations however (e.g. whole blood).

Investigating the 7 site-specific cancer outcomes evaluated in our primary analysis provided very weak evidence of an effect based on all multivariable estimates emphasising the importance of matching causal tissues to exposures being instrumented by our approach. Similarly, we used the same adipose- and neural eQTL datasets from our primary analysis to partition a phenotype where these tissues are unlikely be play a functionally important role. We selected psoriasis for this given it is a primiarly immune-associated disease and used a large-scale GWAS from the UK Biobank (n=462,933) to apply our genetic colocalization pipeline. However, this resulted in only 2 and 1 variants with evidence of colocalization using adipose- and brain-tissue derived gene expression respectively, which is not sufficient to derive meaningful conclusions from our approach.

As a final sensitivity analysis, we sought to evaluate the effects where adipose- and brain-tissue instruments provided evidence of an independent effect on cancer outcomes. To do this, we repeated our instrument derivation pipeline using gene expression data derived from whole blood from the eQTLGen consortium study (n=31,684). Colocalization analysis was performed at all 915 BMI loci using summary statistics derived in whole blood. This identified 162 variants where there was evidence of a shared causal effect (PPA4 > 0.8) between BMI and whole blood derived gene expression. Repeat multivariable analyses using the tissue-paritioned approach indicated that the brain-tissue BMI effect on lung cancer, and adipose-tissue BMI effect on endometrial cancer risk remained robust when assessed against whole blood-tissue instrumented BMI (**Table S10**). While we advocate that tissue eQTL datasets used for instrument derivation should be obtained from datasets of comparable sample size to minimize the introduction of bias on the performance of exposures in the model, this analysis provides a useful robustness analysis for the findings presented in the present study.

## Supplementary Figure 1

Effect of varying the PPA4 threshold on instrument strength


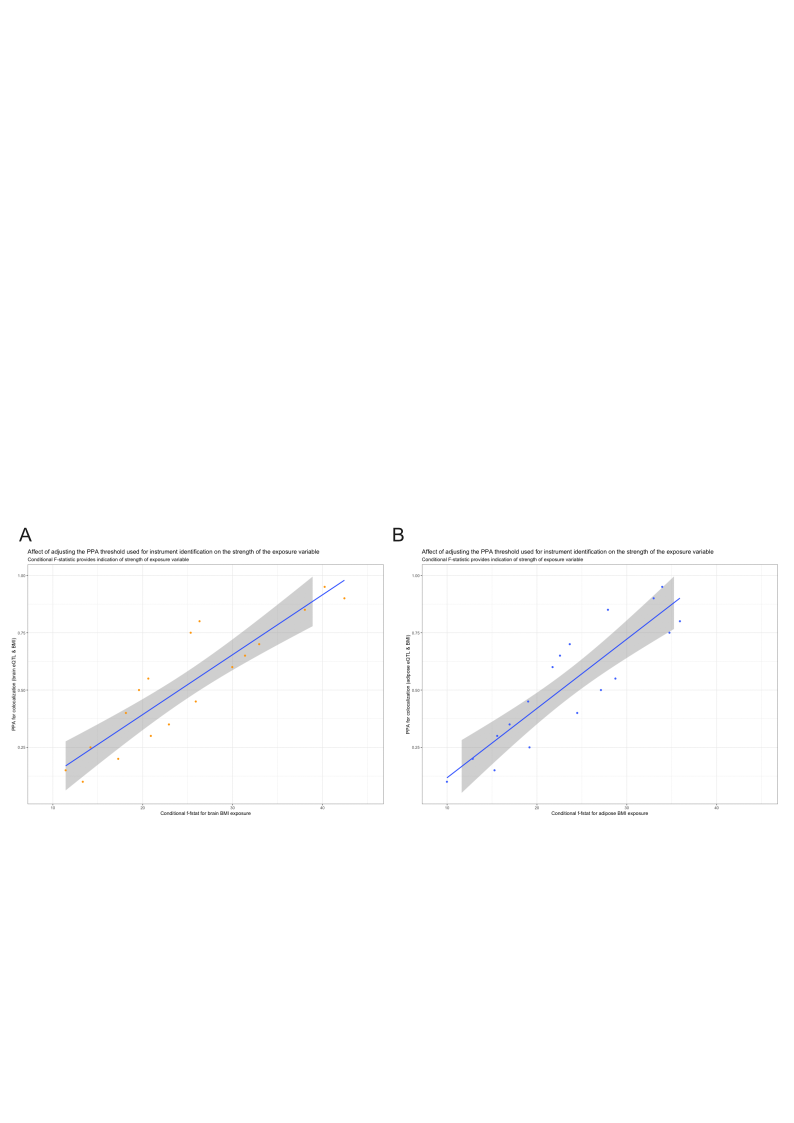


To evaluate the robustness of the PPA4 threshold for instrument derivation, we have conducted an extended analysis to investigate how lowering the threshold would influence the conditional F-statistics in our analysis with coronary artery disease as our outcome. In conclusion, we found that lowering the PPA4 threshold for our approach results in overall lower conditional F-statistics for our exposures, therefore suggesting that the initial cut-off of PPA4 proposed is likely the most appropriate for our method:

## Supplementary Figure 2

Simulation analyses

##
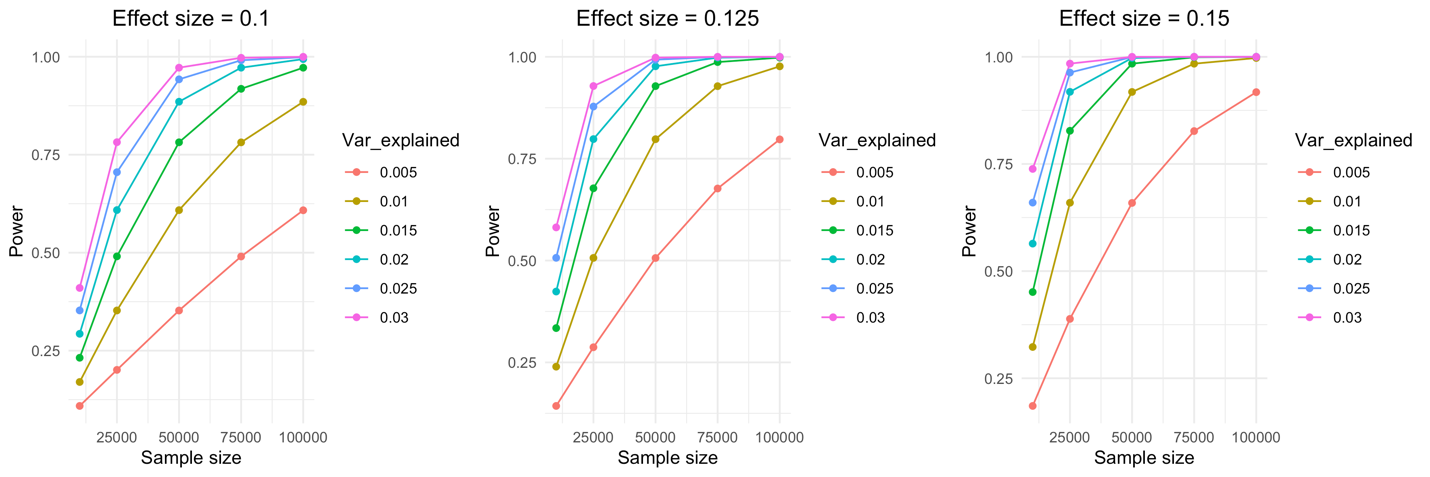


Simulations were conducted using to investigate how variance explained by genetic instruments in our study contributes to statistical power across various sample sizes. Simulations were conducted using the ‘*simulateGP*’ R package to evaluate the relative power needed for instrument derivation across a range of effect sizes (0.1, 0.125 & 0.15), outcome sample sizes (10,000, 25,000, 50,000, 75,000 & 100,000) and proportion of variance explained by tissue-partitioned instruments (0.5%, 1%, 1.5%, 2%, 2.5% & 3%) derived from a simulated GWAS of n=700,000 with a pool of 915 independent genetic instruments (based the BMI GWAS by Yengo used in our applied analysis). Simulations suggest that our approach is adequately powered as long as tissue-partitioned instruments explained at least 1% of the variance in the exposure trait, as well as analysing outcome GWAS datasets consisting of at least 75,000 participants.

# References

1. Yengo L, Sidorenko J, Kemper KE, Zheng Z, Wood AR, Weedon MN, et al. Meta-analysis of genome-wide association studies for height and body mass index in ∼700000 individuals of European ancestry. Human molecular genetics. 2018;27(20):3641-9.

2. Purcell S, Neale B, Todd-Brown K, Thomas L, Ferreira MA, Bender D, et al. PLINK: a tool set for whole-genome association and population-based linkage analyses. Am J Hum Genet. 2007;81(3):559-75.

3. Kibinge NK, Relton CL, Gaunt TR, Richardson TG. Characterizing the Causal Pathway for Genetic Variants Associated with Neurological Phenotypes Using Human Brain-Derived Proteome Data. Am J Hum Genet. 2020;106(6):885-92.

4. Qi T, Wu Y, Zeng J, Zhang F, Xue A, Jiang L, et al. Identifying gene targets for brain-related traits using transcriptomic and methylomic data from blood. Nat Commun. 2018;9(1):2282.

5. Battle A, Brown CD, Engelhardt BE, Montgomery SB. Genetic effects on gene expression across human tissues. Nature. 2017;550(7675):204-13.

6. Fromer M, Roussos P, Sieberts SK, Johnson JS, Kavanagh DH, Perumal TM, et al. Gene expression elucidates functional impact of polygenic risk for schizophrenia. Nat Neurosci. 2016;19(11):1442-53.

7. Ng B, White CC, Klein HU, Sieberts SK, McCabe C, Patrick E, et al. An xQTL map integrates the genetic architecture of the human brain's transcriptome and epigenome. Nat Neurosci. 2017;20(10):1418-26.

8. Grundberg E, Small KS, Hedman Å K, Nica AC, Buil A, Keildson S, et al. Mapping cis- and trans-regulatory effects across multiple tissues in twins. Nature genetics. 2012;44(10):1084-9.

9. Consortium G. The GTEx Consortium atlas of genetic regulatory effects across human tissues. Science. 2020;369(6509):1318-30.

10. Zhu Z, Zhang F, Hu H, Bakshi A, Robinson MR, Powell JE, et al. Integration of summary data from GWAS and eQTL studies predicts complex trait gene targets. Nat Genet. 2016;48(5):481-7.

11. Giambartolomei C, Vukcevic D, Schadt EE, Franke L, Hingorani AD, Wallace C, et al. Bayesian test for colocalisation between pairs of genetic association studies using summary statistics. PLoS Genet. 2014;10(5):e1004383.

12. Leyden GM, Shapland CY, Davey Smith G, Sanderson E, Greenwood MP, Murphy D, et al. Harnessing tissue-specific genetic variation to dissect putative causal pathways between body mass index and cardiometabolic phenotypes. Am J Hum Genet. 2022.
